# Approaches for Reactive Oxygen Species and Oxidative Stress Quantification in Epilepsy

**DOI:** 10.3390/antiox9100990

**Published:** 2020-10-14

**Authors:** Rhoda Olowe, Sereen Sandouka, Aseel Saadi, Tawfeeq Shekh-Ahmad

**Affiliations:** The Institute for Drug Research, The School of Pharmacy, Faculty of Medicine, The Hebrew University of Jerusalem, Jerusalem 91120, Israel; rhoda.olowe@mail.huji.ac.il (R.O.); Sereen.Sandouka@mail.huji.ac.il (S.S.); aseel.saadi@mail.huji.ac.il (A.S.)

**Keywords:** reactive oxygen species, oxidative stress, epilepsy, seizures, lipid peroxidation, protein oxidation, redox status

## Abstract

Oxidative stress (OS) and excessive reactive oxygen species (ROS) production have been implicated in many neurological pathologies, including acute seizures and epilepsy. Seizure-induced damage has been demonstrated both in vitro and in several in vivo seizure and epilepsy models by direct determination of ROS, and by measuring indirect markers of OS. In this manuscript, we review the current reliable methods for quantifying ROS-related and OS-related markers in pre-clinical and clinical epilepsy studies. We first provide pieces of evidence for the involvement of different sources of ROS in epilepsy. We then discuss general methods and assays used for the ROS measurements, mainly superoxide anion, hydrogen peroxide, peroxynitrite, and hydroxyl radical in in vitro and in vivo studies. In addition, we discuss the role of these ROS and markers of oxidative injury in acute seizures and epilepsy pre-clinical studies. The indirect detection of secondary products of ROS such as measurements of DNA damage, lipid peroxidation, and protein oxidation will also be discussed. This review also discusses reliable methods for the assessment of ROS, OS markers, and their by-products in epilepsy clinical studies.

## 1. Introduction

Epilepsy remains one of the most common neurological diseases, affecting over 70 million people worldwide, which imposes enormous physical, psychological, social, and economic burdens on patients, their caregivers, and society [1]. Despite substantial progress in epilepsy research, approximately 35% of all epilepsy patients are resistant to anti-seizure drugs (ASDs) and continue to experience recurrent unprovoked spontaneous seizures [2].

Many of the epilepsies are acquired conditions following an insult to the brain such as a prolonged seizure, traumatic brain injury (TBI), or stroke [3]. It is important, therefore, to understand and identify the key mechanisms involved from the insult to epilepsy to provide prophylactic treatment for preventing epilepsy development in those at risk [4,5].

In recent decades, comprehensive studies on epilepsy are focusing on the process of epileptogenesis: a process during which changes occur in the brain tissue following a precipitating injury that initiates a dynamic sequence of events, including structural, cellular, and molecular changes occurring over time. This eventually leads to the occurrence of unprovoked seizures [6].

It is now well established that oxidative stress (OS) plays a key role in acute neurological injuries such as prolonged seizures [7], stroke [8], and TBI [9] as well as in neurodegenerative diseases such as Parkinson’s [10] and Alzheimer’s disease [11,12]. In epilepsy, it has been shown that reactive oxygen species (ROS) and induction of OS are common sequelae that occur following a brain insult and contribute to neuronal death and the development of the disease [13]. Most recently, animal studies have addressed the role of OS and ROS generation in sustaining status epilepticus (SE) and generating spontaneous seizures in chronic epilepsy [14,15,16]. Furthermore, neuroinflammatory responses, which is considered as a normal response in maintaining homeostasis and possibly enabling the central nervous system (CNS) to cope with insults induced by increased neuronal activity, have been shown to be functionally interconnected with OS. These two phenomena contribute to the neuropathology of seizures both in human and animal models of epilepsy [17,18,19]. Moreover, human data had largely confirmed the animal studies. OS markers were detected in the hippocampus of humans who died following SE or have chronic pharmaco-resistant epilepsy [20].

Notwithstanding the above, previous results from clinical trials using various antioxidants, including vitamin E, tirilazad, N-acetylcysteine, and ebselen, have been contradictory [21,22,23,24,25]. A potential reason for these mixed results includes failure to appreciate and characterize the individual unique oxidative processes occurring in different diseases. Moreover, therapy with antioxidants may need to be given early in chronic insidious neurologic disorders to achieve an appreciable clinical benefit. Other factors limiting the efficacy of antioxidants are the poor penetration of the blood-brain-barrier (BBB) and short-lived neuroprotective effects [26]. Therefore, direct and accurate measurement of ROS and OS markers may provide a better understanding of the molecular mechanisms whereby they can contribute to pathological changes, including changes that occur during epileptogenesis, which may eventually lead to the emergence of spontaneous seizures, and, hence, to provide a more appropriate intervention to halt, or at least to modify the development of epilepsy.

Until presently, there are no sensitive, facile, and accurate assays to measure OS markers, which may translate to therapeutic purposes. In addition, designing the more specific and targeted redox modulations requires specific subcellular compartments for oxidations to be explored. ROS quantification, and especially measurement of radicals, in wet samples such as biopsies, water solutions, and cell cultures, using common techniques is usually associated with a lack of accuracy. In addition, detected levels of specific ROS may be influenced by a large number of experimental parameters. However, it is sometimes desired to observe even very small differences in the generation of ROS between different biological samples. 

In this review, we discuss the most common approaches and assays used in preclinical and clinical studies for quantification of ROS and OS markers in the field of epilepsy research.

## 2. Sources of ROS in Epilepsy

In the emerging field of OS studies, imbalance in ROS resulting from an increase in free radicals production and decrease in available antioxidants, consequent neuronal cell damage and death have been well documented in the initiation and progression of several neurologic diseases including in seizures and epilepsy [27,28,29,30,31,32]. However, whether excessive ROS production in the neurons is a cause or consequence (or maybe both) of seizure activity, the intriguing question is which source of ROS is involved in such an activity. Since ROS measurement from seizure-like activities is commonly performed in tissue or cell homogenates, extracellular fluids or brain regions do not show a clear demonstration of the ROS origins [31,33,34]. 

Many studies have suggested that mitochondria are a major source of ROS generation during seizure activity [30,35]. It has been reported that the major ROS production site in brain tissue has been attributed to complex I of the mitochondrial respiratory chain [34,36,37], and yet suggested that this particular site is unlikely to be involved in seizure-related ROS generation; since the increase in energy load or Ca^2+^ concentration by the process of oxidative phosphorylation has been shown to cause a strong suppression of complex I-dependent superoxide formation [38,39]. 

Malinska and colleagues [30] studied the potential contribution of complex III dependent superoxide production in seizure-induced ROS generation of isolated rat brain mitochondria. In order to distinguish the complex III-dependent superoxide generation from other possible sites, the bc_1_ complex inhibitor antimycin was used since it inhibits respiration and dissipates the mitochondrial membrane potential by effectively blocking the reverse electron flow-driving superoxide production by complex I. It has been documented that this particular condition allows for the identification of the possible maximal contribution of complex III to ROS production. They further reported that elevated concentration of Ca^2+^ influx into the cytosol apparently diminished the reverse and direct electron flow-driven ROS generation by complex I. They then proposed that complex III-dependent superoxide generation is an important trigger point, which can directly activate cytosolic signal transduction processes requiring a superoxide in seizure-like events [40,41]. However, in the process of epileptogenesis, the potential inducer for this complex cascade of events is not yet known.

In addition to the mitochondrial ROS production, burgeoning evidence from recent studies have suggested other alternative sources of ROS, which are essential enzymes speculated to be playing a pivotal role in seizure-induced ROS generation including NADPH oxidase and xanthine oxidase (XO) that have been identified to be activated by N-methyl-D-aspartate (NMDA) receptor. However, their contribution to excitotoxicity during seizure activities remains unclear [42]. Girouard and colleagues [43] reported that, following a Ca^2+^-dependent pharmacologic activation of NMDA receptors, acute activation of NADPH oxidase in neurons was mainly shown [43,44]. Furthermore, activation of NADPH oxidase has been observed during seizure activity [31]. In addition, in increased metabolism such as those occurring during seizures, XO may also represent a major potential source of ROS. In an attempt to understand the contribution of the two enzymes to ROS generation, Kovac et al. studied the NADPH oxidase-dependent ROS generation by inhibiting NADPH oxidase with AEBSF (a well-established inhibitor of NADPH oxidase activation) and inhibiting XO with oxypurinol in a low-Mg^2+^ model of induced seizure-like activity. They reported a significantly lower ROS production following this inhibition, suggesting that the primary sources of ROS generation during neuronal hyperexcitability in this model of seizure-like activity were NADPH oxidase and XO [42]. The studies convincingly showed that NMDA-receptor-dependent ROS production in neurons is dependent on NADPH oxidase activation, pointing that targeting these mechanisms (i.e., by inhibiting NADPH oxidase or XO) is neuroprotective. 

Furthermore, the role of ROS generated by NADPH oxidase by specific inhibition of NADPH oxidase activity with the antioxidant apocynin was evaluated in a pilocarpine-induced Temporal Lobe Epilepsy (TLE) model, and a decreased ROS generation in the hippocampal regions of the brain was observed, indicating that NADPH oxidase dependent OS is involved in SE induced neurodegeneration and conclusively suggested that intensive research on NADPH oxidase inhibitors as well as their mechanism of actions might present a promising target for therapeutic interventions in TLE [31].

## 3. General Methods for ROS Detection and Quantification

Reactive oxygen species (ROS) have been regarded as highly reactive metabolites of oxygen, including superoxide (O_2_^−^), hydrogen peroxide (H_2_O_2_), hydroxyl radical (HO•), and peroxynitrite (ONOO^−^) with a lifetime in biological systems ranging from nanoseconds to seconds depending on cellular antioxidant levels and on their reactivity [45]. Due to their high reactivity and numerous clearance mechanisms, ROS exist in vivo in a very low nanomolar steady-state concentrations, making it difficult to measure [46]. ROS detection in biological systems, therefore, requires probes that can rapidly react with ROS in order to compete with antioxidants and produce stable quantifiable products [45]. A number of direct and indirect methods have been proposed for the detection or quantification of ROS generation [47]. Unfortunately, free radicals involved in OS processes at toxic levels have a very low concentration that detection or quantification of these very short-lived species is extremely challenging [48].

In general, spectrophotometry and fluorometric methods have long been used even until now, as they are based on the alteration of absorption characteristics of chromogens after interaction with ROS and on the principles based on the properties of probes, respectively. Of the mostly employed, electron spin resonance (ESR), which is also known as electron paramagnetic resonance (EPR) spectroscopy, has been reported as the only analytical approach that permits direct detection of free radicals [48]. Unpaired electrons of radical species including nitric oxide, hydroxyl radical, or superoxide are short-lived and too low in concentration to be directly detected by ESR in biological systems. Therefore, ESR measurement of more stable secondary radicals species formed by adding exogenous spin-traps and diamagnetic compounds that, upon reaction with transient primary radical species, give long-lasting radicals that can accumulate to levels permitting detection by ESR, are employed to circumvent this problem [49]. This newly developed spin trap yielding spin adducts with longer lifetimes have made quantitative measurements of free radicals in biological systems possible [50]. The most commonly employed traps are nitrones like Phenyl-*tert*-butylnitrone (PBN), cyclic nitrones like 5,5-dimethylpyrroline-N-oxide (DMPO), or nitroxides such as hydroxyl 2,2,6,6-Tetramethyl-1-piperidinyloxyl (TEMPO) [51,52]. Interestingly, the spin probe techniques provide a relatively safe way of identifying actual species present in the system [53] and also used to measure tissue oxygen consumption and concentration in living tissues where traps usually display a marked selectivity for particular species, permitting modulation of sensitivity toward a given radical [48,54]. The use of spin traps such as PBN has been historic in the detection of organic radical products of lipid peroxidation, while DMPO has been used in the detection of hydroxyl and superoxide radicals [55]. Furthermore, Dikalov et al. studied the use of a new spin trap, hydroxylamine 1-hydroxy-2,2,6,6-tetramethyl-4-oxo-piperidine (TEMPONE-H) to quantify peroxynitrite, superoxide, and peroxyl radicals in biological systems and showed that the hydroxylamine TEMPONE-H reacts with these radicals, forming a stable nitroxide radical TEMPONE. They also reported that the TEMPONE-H sensitivity in the detection of peroxynitrite and superoxide nitrite are about 10-fold higher than using spin traps DMPO [50]. The major limitation of this method is the availability of specific and expensive equipment, which is not usually present in biological laboratories [47].

## 4. Quantification of the Superoxide Anion

The superoxide anion (O_2_^−^), the first radical species produced by mitochondria and a progenitor for the formation of many other ROS, including H_2_O_2_, ONOO^−^, and lipid peroxides, is one of the key ROS in biological systems playing a pre-eminent role in biology and pathophysiology since it is formed by many mammalian enzymes with significant biological reactivity [45,56]. As a result of superoxide radical reactivity with other compounds and its spontaneous and enzyme-driven dismutation, its detection is generally more strenuous than that of H_2_O_2_ [56]. Here, we mention few procedures from spectrophotometric methods and procedures that have been introduced in the last few years that are generally much more sensitive than previous and that involve the use of fluorescence probes. 

### 4.1. Nitroblue Tetrazolium Assay

Nitroblue tetrazolium (NBT) is a nitro-substituted aromatic that can be reduced by O_2_^−^ through a one electron transfer reaction, yielding a partially reduced monoformazan (NBT^+^) as a stable intermediate, where its formation can be monitored spectrophotometrically at 550 to 560 nm [57]. Armstead et al. [58] studied the superoxide anion generation in bicuculline-induced seizures in piglets. The superoxide dismutase (SOD)-inhibitable NTB reduction was determined as an index for superoxide anion production. The concentration of nitro-blue formazan was determined spectrophotometrically at 515 nm. The result of the study showed that SOD-inhibitable NTB reduction was increased in piglets subjected to bicuculline-induced seizures for 20 min when compared to control animals [58].

### 4.2. Cytochrome c Measurement of Superoxide Anion

In a number of situations to assess the rate of superoxide formation, a reliable method for the measurement of superoxide by detection of cytochrome c, which involves the reduction of ferricytochrome c to ferrocytochrome c has been employed (Equation (1)) [59].

(1) $${\mathrm{Fe}}^{+3}\;\mathrm{cyt}\;c+\;\cdot {O_{2}}^{-}\to {\mathrm{Fe}}^{+2}\mathrm{cyt}\;c+O_{2}$$

Ferricytochrome c reduction has been reported to be a time-honored method accurate enough to detect large amounts of O_2_^−^ released by cells into the extracellular space following various chemical reactions. However, since this method is not completely specific for superoxide [45], the addition of enzyme inhibitors like CN^−^ or scavengers of reactive species such as catalase can minimize any reoxidation. The enzymes are inactivated by superoxides by oxidizing the Fe(II) moiety from its cubane cluster. Then the superoxide concentration can be estimated by the degree of enzyme inactivation. This reaction can be monitored calorimetrically at 550 nm. For example, aconitase catalyzes the conversion of citrate to isocitrate, following the conversion of 20 mM isocitrate to cis-aconitate using absorbance at 240 nm. The activity of the enzyme can be monitored [57].

### 4.3. Chemiluminescence Detection of Superoxide Anion

Another fondly used method for measuring superoxides is the chemiluminescence technique with a widely used Lucigenin-induced chemiluminescence. In general, because of the limited permeability, this method is more applicable to cell-free assays for intracellular superoxide anion [60]. Chuang et al. [61] measured the superoxide anion levels determined by lucigenin-enhanced chemiluminescence in the hippocampal CA3 subfield of kainic acid (KA)-induced SE in rats, and reported a significant upsurge in superoxide anion levels, which took place as early as 3 h after the induction of experimental temporal lobe SE. The susceptibility of the compound to cause a phenomena known as redox recycling, in which the lucigenin radical reacts with oxygen to generate O_2_^−^ and superoxides are overestimated, has raised a question on the accuracy of the technique [57]. To circumvent this, the use of low concentrations of this compound has been suggested [62]. In an effort to make efficient use of chemiluminescence, other compounds have also been used as chemiluminescence substrates including coelenterazine, which is a lipophilic compound that is brighter than lucigenin and does not have redox recycling. However, coelenterazine-dependent chemiluminescence is not completely specific toward superoxides because peroxynitrite will also result in luminescence in the presence of coelenterazine. To mitigate against these circumstances, selective use of ONOO^−^ scavengers may aid in the separation between the contributions of superoxides and peroxynitrite to chemiluminescence [45,57].

### 4.4. Fluorescence Analysis of Superoxide Anion with Dihydroethidium

**Figure 1:**
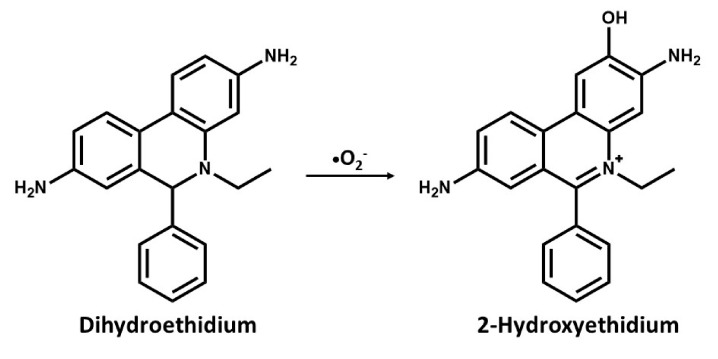
Chemical structures and oxidation reaction of Dihydroethidium to 2-Hydroxyethidium by the superoxide anion.

In a recent procedure based on the change in fluorescence of a probe used for DNA staining ethidium bromide (E^+^) has been reported to be effective in the detection of superoxides. The use of dihydroethidium (DHE) or hydroethidine (HE), which is a reduced form of E^+^ has been used as a selective probe for detecting superoxides and is specifically oxidized by superoxide anions [63,64]. In cell suspensions, intracellular oxidation of HE to ethidium by superoxides has been analyzed with flow cytometry [65], while cellular and regions displaying increased rates of oxidant production can be visualized with digital imaging microfluorometry in vivo [66]. In an attempt to detect seizure-like events in rat hippocampal slices cultures, Malinska et al. [30] measured the rate and site-specific superoxide generation in isolated rat hippocampal mitochondria by determining the SOD-sensitive oxidation of the superoxide-specific fluorescent dye, hydroethidine to hydroxyethidine, and E^+^. They reported a complex III dependent superoxide production. However, the long-held notion was that superoxide reacts with HE to form E^+^, but it has been discovered that superoxide reacts with HE to form a fluorescent product that is distinctly different from E^+^, which binds to DNA and leads to the enhancement of fluorescence [64]. As with dihydroethidium, this compound intercalates with mitochondrial DNA resulting in red fluorescence, suggesting that HE reacts fairly rapidly with O_2_^−^ forming 2-hydroxyethidium cation (Figure 1), which is a unique red fluorescent marker product[67,68]. This has become one of the most frequently used probes for measuring cellular superoxide [69,70].

Previous methods for the measurement of superoxide production with mitochondrial preparations have been reexamined as reports have shown that measurement of superoxides anion can be hampered by lack of an assay sensitive enough to compete with endogenous SOD and, at the same time, selective for superoxides [56,67]. In lieu of this, Zhao et al. [64] reported that, in other to specifically detect superoxides with hydroethidium, the hydroxylated product should be distinguished from E^+^ using high-performance liquid chromatography (HPLC) [67]. However, separating the two products by fluorescence microscopy is difficult as there is an overlap in the emission spectra. Following the weakness of this method, a modified DHE analogue called mitoSOX has been developed to allow the detection of superoxides within the mitochondria of live cells [71]. mitoSOX has been said to react with O_2_^−^ to form 2-hydroxy-mito-ethidium (2-OH-Mito-E^+^), which can be detected and quantified using HPLC or mass spectrometry to provide a more reliable method for superoxide production [68,72]. Thus, the mitochondria-targeted probe, Mito-SOXRed forms 2-OH-Mito-E^+^ in the presence of superoxide. While Zielonka et al. [73] reported that accurate measurement of 2-OH-Mito-E^+^ can be achieved through the use of HPLC, and, to better quantify superoxides, mitoSOX has been used with flow cytometry [74], and mitochondrial O_2_^−^ was reported to be accurately quantified in live cells using selective excitation of 385–405 nm and detection at an emission of 560 nm [67]. The main assays for direct quantification of the superoxide anion are listed in Table 1.

## 5. Quantification of Hydrogen Peroxide

**Figure 2:**
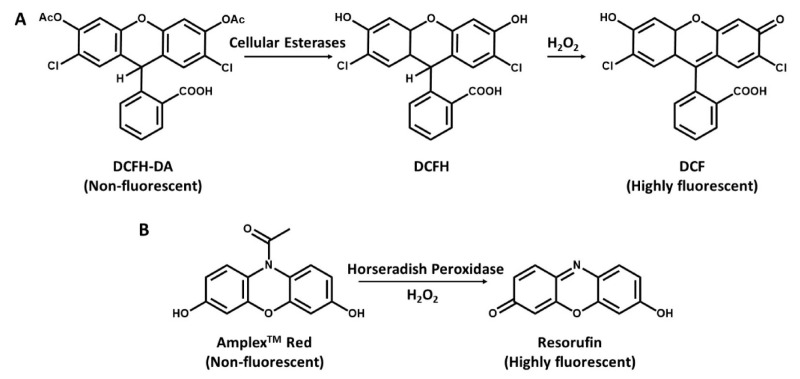
Detection of hydrogen peroxide with DCFH-DA and N-acetyl-3,7dihydroxyphenoxazine (Amplex Red). (**A**), DCFH-DA is cleaved by intracellular esterases to DCFH and oxidized by ROS to the highly fluorescent molecule DCF. (**B**), Conversion of Amplex Red to highly fluorescent Resorufin in the presence of H_2_O_2_ and horseradish peroxidase (HRP).

Virtually among all the reactive oxygen species, hydrogen peroxide (H_2_O_2_) is the most stable and abundant, mainly because it is the by-product of SOD, which is the superoxide scavenging enzyme. Detection of H_2_O_2_ is also possible by the fluorescence method. Researchers generally prefer measurements of H_2_O_2_ when studying mitochondrial ROS in live cells. One good reason for this preference is that there is an increasing variety of probes that can detect cellular H_2_O_2_ [56]. Another reason is that it is the most important ROS with regard to cell cycle regulations. Measurement of hydrogen peroxide involves the use of certain fluorogenic substrates, serving as a hydrogen donor used in conjunction with the horseradish peroxidase (HRP) enzyme to produce a fluorescence product (Figure 2) in which the amount of fluorescence products increases with the increasing amount of hydrogen peroxidase [49]. Also noted by Zhou et al. [75] that hydrogen peroxide detection under the action of HRP uses several substrates for the fluorometric quantification, and it is measured either by an increase or a decrease in the fluorescence of the substrate upon oxidation. Among these substrates are diacetyldichloro-fluorescein [76], Amplex Red [75], scopelectin [77], and homovanillic acid (HVA) [78]. The more commonly used fluorogenic substrates that have been used in conjunction with HRP to produce intensely fluorescent products in assays for hydrogen peroxide quantification are discussed below and summarized in Table 1.

### 5.1. Quantification of Hydrogen Peroxide with DCF 

The most widely used method for measuring cellular production of H_2_O_2_ is a fluorescence-based method that has been earlier introduced in literature and has a good application to ROS measurements in mitochondria. It is based on 2′,7′- dichlorofluorescein (DCFH) oxidation. A probe with a low basal fluorescence but, when oxidized in the presence of H_2_O_2_, it is converted into 2′,7′- dichloro-fluorescein (DCF), a highly fluorescent component with 500 nm excitation and 520 nm emission [79]. The dictate form of DCFH and DCFH-DA is a cell-permeable ester, taken up by the cells and is hydrolyzed to form DCFH, which remains trapped intracellularly (Figure 2A) [49,80]. A major interest of this method is its simplicity, albeit it may be an indicator of cellular redox status rather than a real estimation of intracellular H_2_O_2_ production as it is limited by low specificity [81]. However, irrespective of the fault related to cell leakage and probable intrusion by cytochrome c and other iron proteins [81,82], DCFH-DA measurements still provides robust and reliable measurements of mitochondrial production of H_2_O_2_ in cells [83,84,85].

### 5.2. Quantification of Hydrogen Peroxide with Amplex Red 

Second to the DCF is the N-acetyl-3,7-dihydroxyphenoxazine (Amplex Red), which can be used to selectively detect H_2_O_2_ released from isolated mitochondria [86], it is oxidized by H_2_O_2_ in the presence of horseradish peroxidase (HRP) and then converted to resorufin [75]. The Amplex Red assay is based on the HRP-catalyzed oxidation of the non-fluorescent molecule Amplex Red to resorufin (Figure 2B), which, upon excitation at 530 nm, emits light at 590 nm [45,81]. However, since the production of hydrogen peroxides is dependent on the quick dismutation of superoxide, the presence of substantial fluxes of superoxide can react with the basal or compound 1 state of peroxidase, leading to the formation of compound III. This reaction can alter the stoichiometry of H_2_O_2_ detection and, therefore, affects its quantitative measurement by Amplex Red [45]. Dikalov et al. [87] reported that a main challenge with the Amplex Red method is its inability to directly assess intracellular H_2_O_2_, while Zhao et al. [88] also reported that the method is susceptible to auto-oxidation of Amplex Red dye and is light sensitive. Due to instability of the dye, at high concentrations (50 µM), it can be autoxidized to O_2_^–^ and H_2_O_2_ to minimize this problem. Low concentration (10 µM) of Amplex red is required [45].

### 5.3. Quantification of Hydrogen Peroxide with Scopoletin

For scopoletin, in the presence of HRP and H_2_O_2_, the fluorescent compound is oxidized to a non-fluorescent reduced derivative. However, it is a specific method based on fluorescence decrease, i.e., a very inaccurate determination [89]. The suitability of scopoletin in the detection of mitochondrial H_2_O_2_ was investigated and it was found that the concentration of 5µM scopoletin is optimal for calibration of the H_2_O_2_ range of interest. However, full linearity was not obtained. It was also reported that a fluorescence decrease is taken as the evidence of scopoletin oxidation through H_2_O_2_ in the presence of HRP [77]. 

### 5.4. Quantification of Hydrogen Peroxide with Homovanillic Acid

Another substrate used for hydrogen peroxidase detection following a reaction with HRP is the homovanillic acid (HVA). This assay is based on the oxidation of HVA into highly fluorescent dimer, mediated by HRP, and dependent on H_2_O_2_. The use of HVA has been regarded as a simple and sensitive fluorometric assay for the determination of H_2_O_2_ produce by neutrophils and macrophages [78]. Like scopoletin, light emission of the fluorescent HVA (oxidized form) was decreased in the mitochondria [77]. Herrero and Barja [90] reported that HVA-related light emission, which is increasingly applied for the detection of mitochondrial H_2_O_2_ was found to interfere with mitochondrial components in the resonance with the wavelength of light emitted from the indicator. 

When using HRP catalyzed substrates to quantitate H_2_O_2_, there are several issues that one should be aware of. For instance, cellular compounds such as thiols can serve as a substrate for HRP and endogenous catalase activity can artificially reduce the amount of H_2_O_2_ present. Depending on the excitation and emission wavelengths, these cellular components can affect the fluorescent signal as with homovanillic dimer. 

## 6. Quantification of Peroxynitrite

### 6.1. Quantification of Peroxynitrite with Dihydrorhodamine

**Figure 3:**
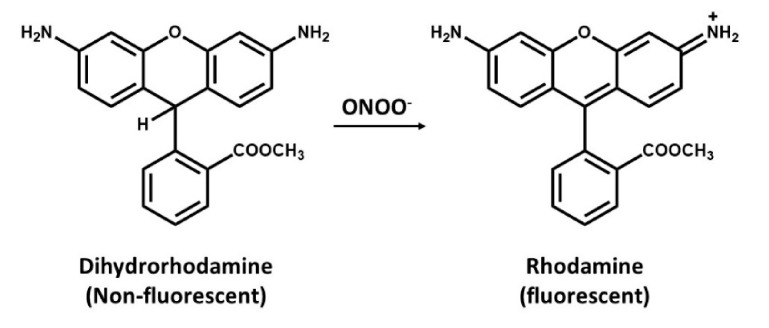
Oxidation of dihydrorhodamine (non-fluorescent molecule) to rhodamine (a fluorescent probe) by peroxynitrite.

Dihydrorhodamine (DHR) is a cell-permeant, commonly used for detection of peroxynitrite (ONOO^−^) [91]. This assay is based on the oxidative conversion of DHR to its corresponding two-electron oxidized fluorescent product, rhodamine (Figure 3). Peroxynitrite readily oxidizes DHR, of which the oxidized rhodamine tends to be retained within the cell. However, peroxynitrite is not only responsible for this oxidation as several cell-derived oxidation and intermediate of DHR are also capable of oxidizing DHR leading into a false negative data. It was then concluded that DHR can only be used as a nonspecific indicator of peroxynitrite [92,93].

### 6.2. Boronate-Based Measurements of Peroxynitrite

**Figure 4:**
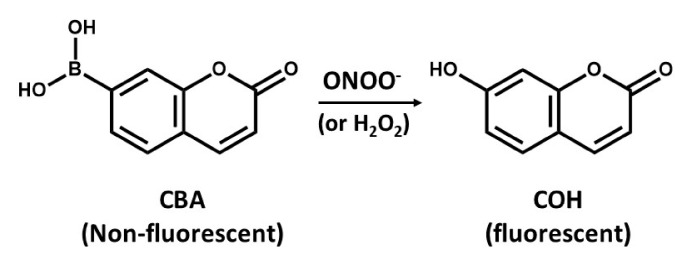
The conversion of non-fluorescent coumarin-7-boronic acid (CBA) into the fluorescent product 7-hydroxycoumarin (COH).

The detection of ONOO- by boronate-based fluorescent probes that have been introduced by Miller and colleagues [94] is similar to that of DHR. Boronate probes have been recently described to react stoichiometrically with ONOO-, yielding the corresponding phenols [95,96]. An example of such a probe is the coumarin-7-boronic acid (CBA), which react rapidly with ONOO^−^ with a rate constant of 1.1 × 10^6^ M^−1^s^−1^ to produce the fluorescent product 7-hydroxycoumarin (COH) (Figure 4) [97]. However, there is a potential problem related to the lack of specificity of boronate probes that can detect either ONOO^−^ or H_2_O_2_, as it seems that reaction of ONOO^−^ with boronate leads to site-specific nitration of an aromatic moiety that can be followed by either HPLC or mass spectrometry [95]. However, it is more likely that boronate probes will provide a more reliable approach for detection of OONO- than using DHR assays [95]. However, the use of ONOO^−^ scavenger such as uric acid might help increase the specificity of boronate probes, but, to validate such approaches, further studies are needed.

## 7. Quantification of the Hydroxyl Radical

The Hydroxyl radical (HO•) is an extremely short-lived oxidant that reacts at near-diffusion limited rates with a wide variety of biological macromolecules has been considered the most toxic ROS[98], as it is directly implicated in OS leading to the possible development or progression of numerous neurodegenerative processes [35,99]. The detection of the hydroxyl radical produced from Fento’s reactions was studied by the application of both 1,2-benzopyrone (coumarin) and 5,5-dimethyl-1-pyrroline-N-oxide (DMPO) as probes to measure hydroxyl radicals by fluorescence and ESR, respectively [100]. Measurement of hydroxyl radicals with fluorescent methods have been reported to be difficult and an indirect method requires the separation of fluorescent radicals by HPLC [101].

## 8. Quantification of Lipid Peroxidation

Lipid peroxidation is a key indicator of OS and one of the most widely used indicators of free radical formation [102]. In general, lipid peroxidation is the conversion of fatty acids in the lipid bilayer to reactive species, as the presence of a large amount of polyunsaturated fatty acids within the inner membrane matrix of the mitochondria makes them susceptible to lipid peroxidation by generating ROS, resulting in neurodegeneration [103]. Since an ultimate noxious product of oxygen radicals that impairs mitochondrial and cellular function by fundamentally damaging the membranes is lipid peroxidation, it is, therefore, of imminent importance to obtain its detailed estimates [104]. Assessments of lipid peroxidation have relied on the measurement of major end products, which are the free aldehydes generated including malondialdehydes (MDA), 4-hydroxy-*trans-*2-nonenal (HNE), Isoprostanes (IsoPs), and Acrolein [105]. 

### 8.1. Malondialdehyde

Malondialdehyde (MDA) is regarded as the most abundant and stable end-product of lipid peroxidation-specific aldehyde, measured as an index of OS. Measurement of MDA has long relied on the detection of thiobarbituric reactive substances (TBARS) in fluid samples like plasma [49]. Since it is the most frequently used method, this reaction is based on detection of secondary reaction products, which results in an adduct that can be measured colorimetrically at 532 nm or by fluorescence using a 530 nm excitation and 550 nm emission wavelengths [106]. A number of studies have reported an increase in MDA levels in plasma samples of epileptic patients and patients with other neurological diseases. In the blood of epileptic patients, the level of MDA increased by 1.4 times, depending on the severity of the disease [107]. Furthermore, since the brain has high lipid content and oxygen consumption and the oxidative metabolism make it susceptible to OS, Menon et al. [108] investigated the effect of epilepsy on lipid peroxidation by studying the MDA levels in epilepsy patients and reported significantly higher MDA levels in these patients compared to the control group. However, since this method remains very sensitive, one drawback of the method is that it is not necessarily specific to MDA [109].

### 8.2. Isoprostanes

Isoprostanes (IsoPs) have been recently reported to be a unique series of prostaglandin-like compounds formed from free-radical-catalyzed peroxidation of arachidonic acid independent of the cyclooxygenase enzyme [110,111]. The discovery of IsoPs as products of nonenzymatic lipid peroxidation has opened up new areas for investigating more on the role of free radicals in pathophysiological and physiological studies, as it has been documented to be an accurate measure for OS in numerous neurodegenerative diseases [112]. Basically, in previous studies, detectable levels of F_2_-IsoPs have been reported on biological fluids such as plasma and urine [113] and a substantial body of evidence noted that measurement of IsoPs in body fluids of both animals and humans provides a reliable approach to assess lipid peroxidation, which is particularly important in in-vivo OS studies indicating that there is ongoing lipid peroxidation that is incompletely suppressed by antioxidant defenses [114,115]. In this respect, because of the high structural diversity of IsoPs, several methods exist to quantify F_2_-IsoPs using gas chromatographic/mass spectrophotometric (GC/MS) or liquid chromatographic/mass spectrophotometric (LC/MS) approaches [116,117,118,119]. These approaches have been reported to yield quantitative results in a low picogram range, as it is a highly sensitive method to measure Isops. However, its main drawbacks are that it requires substantial capital expenditure and seems to be labor intensive [112]. Another method of choice for measurement involves the use of ELISA-based immunoassays, which are simpler, cheaper, and faster even though there is a poor correlation between different immunoassays, as the value obtained from ELISA often do not correspond to those measured by GC/MS or LC/MS. This results from high variability of the immunoassay [120]. Therefore, the most sensitive and accurate quantitative measurement of IsoPs is the GC/MS involving the use of appropriate deuterium-labelled internal standards [116,121]. Using GC/MS analysis, Patel et al. [122] quantified IsoPs following KA-induced SE in rats and reported a large increase in these stable arachidonic acid-derived prostaglandin products, specifically F_2_-IsoPs early after SE in hippocampal subregions.

### 8.3. 4-hydroxy-trans-2-nonenal (HNE) and Acrolein

4-hydroxynonenal (4-HNE or HNE) and Acrolein are straight-chain lipid hydroperoxides that degrade to form reactive aldehyde species, which are also responsible for the oxidative damage caused by lipid peroxidation and can, therefore, be used as biomarkers for lipid peroxidation [123,124]. However, direct measurements of HNE and Acrolein have been reported to be difficult because they react readily with other cell constituents and are rapidly metabolized. Therefore, the formation of these products is usually assessed indirectly by measuring their covalent protein adducts, since an increase in abundance of these adducts is likely to be reflective of increased OS [125]. Most employed methods for measurement of HNE in tissue and plasma samples are the HPLC or GC/MS [126]. HPLC usually involves the use of aldehyde reactive probes including 2,4-dinitrophenylhydrazine and 1,3-cyclohexandione or other similar chemical probes [127].

Acrolein has been most commonly measured using GC/MS [128]. However, a recent technique involving the use of acrolein–protein adducts [129] and aldehyde-sequestering drugs [130] to indirectly detect the compound via its reaction with biological molecules has been reported. Mounting evidence linking HNE and Acrolein with excitotoxic cascades, leading to neurodegenerative diseases has been documented. Williams et al. [131] reported significantly elevated levels of HNE and Acrolein in the hippocampal brain tissue from patients affected by mild cognitive and early Alzheimer’s disease, which were measured using a sensitive and selective LC-MS/MS and HPLC methodology. In mice subjected to pentylenetetrazol kindling and pilocarpine-induced epileptic seizure models, increased MDA content and HNE levels in the hippocampus were measured [132]. Liquid chromatography with fluorescence detection was used for the analysis of lipid peroxidation markers in rat urine following a chemically induced seizure model. Interestingly, while a significant increase in Acrolein concentration was observed, the results for MDA and HNE were inconclusive [133].

## 9. Quantification of Redox Status

The redox status is widely used in the past few decades to describe the redox phenomena in biological systems. Historically, it has been described as the ratio of the interconvertible oxidized and reduced form of a specific redox couple. Furthermore, the redox state of a redox couple is defined by the half-cell reduction potential and the reducing capacity of that couple where the intracellular redox state reflects the state of this couple [134]. Studying redox potential of a specific compartment or cell can be done using a variety of techniques to identify and quantify major redox couples or redox-sensitive proteins within organelles. For this, various methods exist for the monitoring of redox state changes in cells and these include the measurement of the intracellular concentrations of reduced and oxidized glutathione (GSH and GSSG, respectively), measurement of the intracellular concentrations of NAD(P)^+^ and NAD(P)H [135], and measurement of protein thiols whose reactions drive the critical cellular redox system and constitute a larger active redox pool [136]. It is important to know that the ratio of oxidized and reduced forms provide a reasonable estimate of reduction potential for the [NAD]/[NADH] and [NADP]/[NADPH] couples. In contrast, the absolute [GSH] concentration must be accounted for when estimating reducing the potential for glutathione disulfide [GSSG]/[GSH] couples [134]. So far, HPLC has been employed in the quantification of GSH/GSSG and NAD(P)H/NAD(P)^+^ redox potentials [137,138], while the determination of the redox state of several proteins such as Thioredoxin (Trx), and Trx reductase involves the use of mass spectrophotometry and Western blotting in association with the labelling of free thiols [139,140,141]. Some studies have verified changes in redox potential and decreased levels of ATP during SE, which leads to a collapse in brain energy production and supply [142]. Liang and Patel [143] reported an acute decrease in mitochondrial GSH/GSSG in the hippocampal tissue as early as 8 h and up to 7 days following KA-induced SE. In a recent study, GSH and other specific markers of redox status in the mitochondrion were found to decrease in the hippocampus following pilocarpine-induced SE, which becomes permanently damaged during chronic epilepsy [32]. Therefore, altered mitochondrial and cellular redox status may play an important mechanistic link between acute and chronic stages of epilepsy.

## 10. Quantification of Protein Oxidation

ROS-induced damage to proteins can lead to their structure alterations, increased proteolytic susceptibility, and spontaneous fragmentation, affecting the function of receptors, enzymes, and transport proteins. The side chains of various amino acid residues of proteins are particularly susceptible to various forms of either reversible or irreversible protein post-translational modifications, which can lead to the protein functional impairment [144,145,146]. The analysis of protein oxidation products is more complex than that of other biomolecules (e.g., DNA) as there are many different mechanisms found for ROS interaction with the available 20 amino acids and the fact that protein oxidation products that may be absorbed through diet can confound the measurement of these products in the body. We here summarize the two main assays of protein oxidation used to measure protein oxidation products as potential biomarkers of OS.

### 10.1. The Carbonyl Assay

The carbonyl assay is based on the estimation of protein carbonyl groups that can be formed as a result of glycation of proteins by sugars, binding of aldehydes, or by the direct oxidation of amino acid side chains by ROS. It is the most commonly used biomarker of protein damage. Protein carbonyls represent an irreversible form of protein modification and have been demonstrated to be relatively stable [147,148]. These protein oxidation products are formed early during OS and are a result of non-specific oxidant. Thus, they are considered as a marker of overall protein oxidation [149]. Several methods have been designed for the detection of protein carbonylation including spectrophotometric assays, ELISA assays, in-gel fluorescence detection, and Western blotting [150].

Since spectrophotometric methods are relatively inexpensive and can yield results similar to the significantly more expensive immunochemical methods, it is the most used method for protein carbonyl detection, which, therefore, has been commercialized and available elsewhere by many reputable vendors. Moreover, Western blot and in-gel fluorescence tagging can be used for the detection of carbonylated proteins upon electrophoretic separation. In the KA-induced seizure activity in adult rats, Bruce and Baudry [151] assessed protein oxidation by measuring the concentration of protein carbonyl residues in trichloroacetic acid (TCA) precipitates, and reported a large and sustained increase in protein carbonyl content in piriform cortex and hippocampus as early as 8 h after seizure onset and continued to 16 h. At 2 and 5 days after seizure activity, protein carbonyl levels had returned to near control levels [151]. Interestingly, in genetically epilepsy-prone rats (GEPRs) and models of generalized tonic/clonic epilepsy, the seizure activity induced by KA was positively correlated with increased OS markers such as protein carbonyl in the hippocampus of these rats, indicating a greater seizure sensitivity in response to chemoconvalsants when compared with other rat strains [16].

### 10.2. Advanced Glycation End Products (AGEs)

AGEs result from nonenzymatic glycation reactions to proteins and DNA [152]. Although the formation of AGEs is not dependent entirely on OS, they can be used as potential biomarkers and they can either increase or decrease the OS by increased ROS production or activation of antioxidant enzymes, respectively. Although AGEs have been known to be associated with several diseases including cardiovascular diseases, cancer, and neurological diseases, their use for measuring OS is generally avoided because they are not necessarily ROS-mediated. AGEs can be measured using fluorescence-based assays such as spectrofluorometry and fluorescence-activated cell sorting (FACS) [150]. 

**Figure 5:**
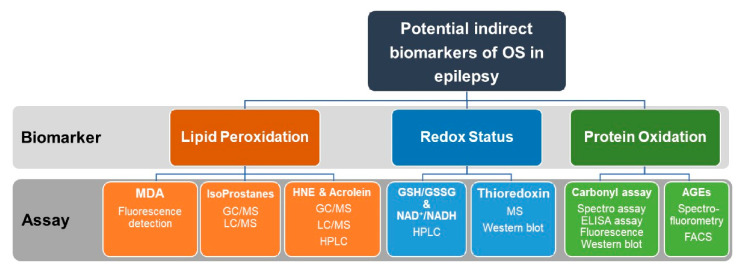
Recommended indirect measurements of oxidative stress (OS) in epilepsy research. Indirect assessment of OS can be achieved by measurements of lipid peroxidation, redox status, and protein oxidation. Lipid peroxidation can be quantified by measuring MDA, Isoprostanes, HNE, or Acrolein. For measurements of redox status, the ratio of GSH/GSSG and NAD/NADPH are assayed. Thioredoxin can be also quantified by MS or Western blotting. Protein oxidation can be quantified by the carbonyl assay or by detection and measuring of AGEs. Assays used for the quantification of the above biomarkers include fluorescence detection, HPLC, GC/MS, LC/MS, ELISA, and Western blotting. OS, oxidative stress. MDA, malondialdehyde. MS, mass spectroscopy. HPLC, high-performance liquid chromatography. GC/MS, gas chromatography/mass spectrometry. LC/MS, liquid chromatography/mass spectrometry. GSH, reduced glutathione. GSSG, oxidized glutathione. NAD^+^/NADPH, Nicotinamide adenine dinucleotide (NAD^+^, oxidized)/(NADPH, reduced).

Commonly used assays for indirect measurements of OS markers including oxidative modifications of lipids and proteins as well as assays for assessments of redox status in epilepsy research are summarized in Figure 5.

## 11. Quantification of Oxidative Stress in Clinical Samples

Evaluation of ROS and OS markers in the clinic is even more challenging than that in experimental in vivo models. Oxidation products of carbohydrates, lipids, proteins, and nucleic acids are routinely measured and considered as biomarkers of OS. In epilepsy patients, these biomarkers are usually performed on peripheral tissues (e.g., plasma, serum, or red blood cells), accurately reflect the OS in the CNS [103]. In clinical practice, epilepsy surgery can often provide access to brain tissue, which may be examined for ROS and oxidative injury markers. Due to the high reactivity of ROS, and, hence, the short half-lives in biological environments, direct measurements of these species are difficult.

In a direct quantitative analysis of ROS, the levels of the superoxide anion were measured in neocortical samples of drug-resistant epilepsy patients, and a significant (~four-fold) increase in its levels was detected in epilepsy patients as compared to controls [153]. In this study, changes in the levels of superoxide were measured by the lucigenin chemiluminescence assay [154]. However, there is controversy whether using lucigenin is appropriate and enough sensitive method for superoxide detection, since lucigenin can result in direct superoxide generation by the probe itself [155,156]. Therefore, it may overestimate the rate of superoxide production. On the other hand, it has been shown that lucigenin cannot take part in redox cycling with molecular oxygen because of its positive one-electron reduction potential [157]. 

GSH is the most abundant intracellular, non-enzymatic low molecular weight antioxidant defense in cells [158]. The ratio of GSH/GSSG is commonly used as a marker of redox status and oxidative stress [159]. Mueller and colleagues [160] investigated the brain GSH levels in patients with focal epilepsy. For this, the researchers developed a technique based on proton magnetic resonance spectroscopy (^1^H-MRS), which was optimized for the detection of coupled cysteinyl compound of GSH [160]. However, the exact absolute concentration of GSH could not be measured due to the complex spin dynamics of the cysteinyl spin system. Therefore, GSH quantification was accomplished by using tissue water content as an internal standard, reflecting the ratio of the signal areas of GSH and water but not the GSH/water ratio [160].

As already discussed, direct detection of ROS in tissue and body fluids is often impractical, particularly in clinical studies. Therefore, indirect measurements of more stable oxidative products are often used for the assessment of oxidative stress. Such oxidative products and markers are usually measured in serum, plasma, erythrocyte, leukocyte, or urine of patients with epilepsy.

Lipid peroxidation products are perhaps the most studied markers of OS in human pathologies, including in epilepsy. The levels of MDA were evaluated in erythrocyte of patients with newly diagnosed idiopathic epilepsy and were found to be significantly lower in those patients as compared to healthy subjects [161]. In this study, lipid peroxidation was evaluated in the erythrocytes of epilepsy patients and healthy individuals using a cellular colourimetric assay by colourimetric detection of the product complex formed by the reaction of malondialdehyde and thiobarbituric acid under a high temperature [162]. The serum thiobarbituric acid reactivity was used in another study to evaluate lipid peroxidation in the serum of epileptic children before and during treatment with valproic acid or carbamazepine [163]. The results showed that, during valproate therapy, the lipid peroxidation levels of the epileptic children increased and the glutathione peroxidase levels decreased in comparison with those levels recorded in the control and pretreatment groups. However, during carbamazepine therapy, lipid peroxidation levels increased when compared with the control group [163]. In another study, the levels of MDA in plasma were measured using the thiobarbituric acid test in epileptic patients with abnormal magnetic resonance imaging (MRI) findings and found to be significantly increased as compared with that of healthy children [164]. In a study by Akarsu et al., the MDA levels were determined in plasma and cerebrospinal fluid patients with febrile and afebrile seizures. While plasma MDA levels were increased in patients experiencing febrile seizures, higher MDA concentrations were detected in cerebrospinal fluid during afebrile seizures, suggesting greater tissue OS in afebrile cases [165].

In an attempt to examine the redox status in the serum of drug-resistant surgically treated epileptic patients, Lopez et al. determined the concentration of products of oxidative damage to lipids by measuring MDA levels as well as the oxidative damage to proteins, by measuring advanced oxidation protein products (AOPP) [166]. For MDA measurements, the same technique based on the reaction between thiobarbituric acid and MDA was used. The measurement of AOPP concentrations, determined by spectrophotometric assay [167], is proposed as a reliable marker to estimate the degree of oxidant-mediated protein damage in vivo since their levels closely correlate with levels of dityrosine, which is a hallmark of oxidized proteins, as well as with pentosidine, a marker of enzymatic protein glycation tightly related to oxidative stress.

Another indirect method to assess OS is the measurements of antioxidant enzymes activity such as SOD, glutathione peroxidase (GPx), and catalase (CAT). Since these enzymes are responsible for the detoxification of different types of ROS, measuring their activity will then reflect the oxidation status. The detoxification of the superoxide anion is carried out mainly by SOD, which catalyzes the dismutation of superoxide anion to hydrogen peroxide. SOD activity is measured using a spectrophotometric assay based on the quantity of the enzyme necessary to achieve a 50% inhibition of pyrogallol autoxidation [168]. While a significant increase in superoxide anion levels was detected in surgically treated patients with refractory epilepsy, there was no significant difference in the SOD activity observed between the patients and healthy controls [153]. The activation of antioxidant enzymes SOD and GPx were investigated in children with epilepsy. While epileptic patients with structural abnormality had a normal activity of SOD, the activity of SOD significantly increased in epileptic children with a negative magnetic resonance image (MRI). The activity of GPx of erythrocytes in all of the epileptic patients was not significantly different from that of normal cases [164]. López et al. investigated the impact of the epilepsy surgery on the activity of SOD, CAT, and GPx in the serum of drug-resistant epileptic patients with TLE [166]. It was found that, compared to control subjects, the epileptic pre-surgery phase presented altered antioxidant enzyme activity. After surgery, the patients showed a tendency to normalization of the studied variables, except for SOD activity. The recovery in GPx activity was also notorious, as it contributes to a decrease in oxidative damage and a better redox balance [166]. 

As already discussed, most of the studies that investigate OS in epilepsy focus on serum lipid peroxide (especially MDA) and erythrocyte antioxidative enzyme activity (especially SOD, GPx, and CAT). Yet, some studied investigated the effects of epilepsy on total antioxidant capacity (TAC). TAC is defined as the “cumulative action of all the antioxidants present in plasma and body fluids.” Thus, this provides an integrated parameter rather than the simple sum of measurable antioxidants [169]. As such, TAC may provide a convenient method for the quick quantitation of antioxidant effectiveness in preventing diseases. Since the antioxidant system has many components, Mahle and Dasgupta [170] suggested that, in clinical practice, it is more convenient to measure TAC of serum for an initial assessment rather than measuring the individual components. Therefore, to test their hypothesis that lipid peroxidation initiated by phenytoin metabolism may be reduced by antioxidants, TAC was measured in serum of epileptic patients receiving phenytoin when compared to normal serum. Epileptic patients were found to have lower TAC in their sera when compared to control subjects [170]. In their study, TAC levels were measured by a method based on the bleaching of the characteristic colour of 2,2′-azino-bis-3-ethylbenzothiazoline-6-sulfonic acid radical cation by antioxidants [171,172]. Using a similar assay to measure TAC levels, Aycicek and Iscan [173] studied the effect of anti-seizure monotherapy on serum TAC in epileptic patients treated with valproic acid, carbamazepine, or phenobarbital. It was found that serum TAC levels were significantly decreased in the untreated group when compared with the controls. Recently, Pauletti et al. reported that OS markers are induced in the hippocampus of humans who died when following SE or with chronic pharmaco-resistant epilepsy [20]. Expanding on these findings, a recently published study confirmed that patients with SE had reduced antioxidants and increased reactive oxygen and nitrogen species. Furthermore, the study also showed that CAT, GSH, and TAC were lower in the SE patients with comorbidity [174].

## 12. Summary

Measurement of ROS and OS following a brain injury that may lead eventually to the development of epilepsy can provide a better understanding of the mechanisms that contribute to the pathological changes, which may help in developing more specific anti-oxidant treatment(s) that can modify the development of epilepsy.

Since ROS are very reactive and have a very short half-life, direct detection of them in tissue and body fluids is often impractical. Therefore, detection of oxidative DNA, proteins, and lipids by-products may be used as biomarkers of OS aimed at preventing disease development. 

When measuring ROS and OS markers in cells and tissues, the rational use of more than one method is recommended for a better testing of the involvement of OS in the studied pathology. The use of various probes to detect ROS and OS markers has been discussed in the above sections of this review. Reaction of ROS with probes may have some implications. For instance, spectrophotometric assays using HRP-coupled reactions have limitations of direct reduction of the oxidized detector molecule by electron transport components, which can affect hydrogen peroxide determination in mitochondria. In like manner, following the susceptibility of ROS to overlap and chemically react with detector molecules, which can result in significant reduction of the steady-state levels of ROS during their measurements, it is suggested that more stable radicals can be formed and detected by adding a wide variety of exogenous spin traps and spin probes such as DMPO, PBN, and TEMPO, which have been used to implicate ROS in various pathophysiological conditions. 

For a high level of ROS production, many initial methods such as cytochrome c reduction and spin trapping were suitable. However, studies of low levels of intracellular ROS require an additional more sensitive approach. For the sake of accuracy, specific detection of superoxides and hydrogen peroxide radicals at low levels should be followed by HPLC and flow cytometer analysis. In addition, detection of peroxynitrite by boronate-based fluorescence can be followed by mass spectrophotometry or HPLC, which are methods commonly employed in research laboratories. Indirect measurement of OS both in preclinical and clinical studies of epilepsy has generally involved the assessment of stable end-products, produced from oxidative processes such as lipid peroxidation, redox status, and protein oxidation. A number of methods, which are helping to fulfil this goal has been validated and includes the use of GC/MS, LC/MS, HPLC, western analysis, ELISA, and FACs. Although, HPLC has been a useful technique for measuring the majority of the potential biomarkers of OS including lipid peroxidation, redox status, and protein oxidation as it is a fairly sensitive and also relatively inexpensive technique. However, though HPLC is valid, it is not as sensitive as LC/MS methods.

Quantification of ROS and studying their fate using advanced analytical techniques will help in developing a broader view of the role of ROS in epilepsy and other diseases. Future progress aimed at developing more accurate and specific assays for ROS and OS quantification will help in a clear understanding of OS involvement in the structural, cellular, and molecular changes that occur over time from injury to the emergence of unprovoked seizures. A better understanding of these changes will help to develop antioxidant therapies not only for the treatment of epilepsy but also for modifying its development.

## Figures and Tables

**Table 1:** Commonly used assays for direct measurement of reactive oxygen species (ROS) in epilepsy.

ROS/Name	Assay	System	Compartment	Limitations	Comments
O_2_^∙−^ Superoxide anion	EPR with spin traps such as PBN, DMPO, or TEMPO	In vivo	Mitochondrial and intracellular system	Expensive and lack of availability of equipment	Spectrophoto-merically at Ex 630 nm Em 650–900 nm
	Cytochrome c	In vivo and in vitro	Mitochondria and intracellular system	Not specific	Absorbance at 550 nm
	Lucigenin-induced chemiluminescence	In vitro	cells	Lucigenin is susceptible to redox recycling	
	DHE	In vitro and In vivo	Mitochondria and intracellular system	Lack of selectivity to the fluorescent dye.	Fluorescence at Ex 470 nm, Em 585 nm
	MitoSOX Red	In vivo	Mitochondria	Better quantification involves the use of flow cytometry	Ex 385–405 nm, Em 560 nm
H_2_O_2_ Hydrogen peroxide	DCFH	In vivo and In vitro	Mitochondria and intracellular system	Low specificity, intrusion by cytochrome c	Ex 500 nm, Em 520 nm
	Amplex red	In vivo	Mitochondria	Auto-oxidation, Inability to directly access intracellular H_2_O_2_	Ex 530 nm, Em 590 nm
HO∙ Hydroxyl radical	EPR and Flouresence assay	In vivo and In vitro	Mitochondria and intracellular system	Measurement by Florescence method is difficult, requires separation of fluorescent radicals by HPLC	Ex 340 nm, Em 456 nm
ONOO^−^ Peroxynitrite	DHR and Boronate probes	In vivo and In vitro	Mitochondria and intracellular system	Lack of specificity, probes undergo nonspecific oxidation in the presence of other oxidants	Ex 510, Em 567

EPR, Electron Paramagnetic Resonance. PBN, Phenyl-tert-butylnitrone. DMPO, 5,5-dimethylpyrroline-N-oxide. TEMPO, hydroxyl 2,2,6,6-Tetramethyl-1-piperidinyloxyl. DHE, Dihydroethidium. DCFH, 2′,7′-dichlorofluorescein. DHR, Dihydrorhodamine.
